# A new approach to dispersing and stabilizing graphene in aqueous nanofluids of enhanced efficiency of energy-systems

**DOI:** 10.1038/s41598-020-64600-5

**Published:** 2020-05-07

**Authors:** H. Hassanloo, S. Sadeghzadeh, R. Ahmadi

**Affiliations:** 1 Energy Department, School of Advanced Technologies, Iran University of Science and Technology, Tehran, Iran; 2 Nanotechnology Department, School of Advanced Technologies, Iran University of Science and Technology, Tehran, Iran

**Keywords:** Energy storage, Mechanical and structural properties and devices, Computational methods

## Abstract

Owing to its severe hydrophobicity, graphene (G) as on dispersed in a fluid usually deposits therein after a short interval of time. Understanding the G-behavior and the factors affecting its deposition could pave a way for creating a substantially stable nanofluid (NF). In this work, a novel method of stabilizing a G-NF is described with selective examples. The results can be extended to develop the science and technology of G-NFs in general. Electrohydrodynamic forces are used as a controlling factor in the presence of magnetic nanoparticles (MNPs). Contrary to common chemical methods employed for preparing G-NFs, which depend on establishing bonds between the components, the physical method introduced in this article could be used as a novel approach not only to dispersing G in a fluid carrier but also to resolve the common problems originating from utilizing such chemical methods as increasing thermal resistance through adding various types of surfactants. The effects of various factors on the stability of the G-NFs are described. By increasing 50%, 100% and 170% of G, the G sitting rate increased by 43%, 82%, and 109%, respectively. With the addition of one, two and three layers to a G-monolayer, the G sitting rate grew by 77%, 153%, and 263%, respectively. Further, the G-behavior in the presence of MNPs and varied intensive electric fields were studied to optimize an electric field that could stabilize a single-layer G sheet in aqueous NFs. Adding MNPs promptly stabilizes a water/ethylene glycol/G NF in an applied electric field of 0.05 V/Å.

## Introduction

Producing fluids with improved thermophysical properties has been a challenge for researchers for many years^1,2^. There are several reports on nanoparticles (NPs) reinforced nanofluids (NFs) disregarding challenges concerning dispersion of NPs in a suitable liquid carrier^1–3^. The advent of nanomaterials with improved properties, particularly higher thermal conductivity, enables researchers to develop NFs with improved thermophysical properties. Graphene (G), an important member of the carbon nanomaterials^3^, is made of C-atoms arranged in a two-dimensional (2D) hexagonal network, where each atom having a strong covalent bond with other three C-atoms of an average 1.42 Å bond length. Having the highest thermal conductivity among different NPs, G could be considered as one of the prime candidates to be combined with conventional heat-transfer fluids to obtain NFs with higher thermophysical properties^4–12^. Researches have outlined the advancement on preparation and assessment methods and the techniques to enhance the stability of G-NFs and outlook prospects. The strong hydrophobicity of G and the influence of gravitational forces are considerable obstacles to producing such NFs with long-term stabilities. Several methods are employed to disperse not only G but also other NPs in aqueous solutions^13,14^. One such technique is adding surfactants and the other being modifying the G surfaces, which could reduce the thermophysical properties of the prepared NFs, although they hardly produce long-term stable NFs. For instance, Kim *et al*.^15^ found that a blend of dodecyl betaine (DB) surfactant and a 0.1 wt% G in water, with a ratio of G: surfactant greater than 1:1, not only does not improve the stability but also makes it more unstable compared to that having no any surfactant.

Shazali *et al*.^16^ showed that hydrofurfuryl polyethylene glycol-functionalized G-NPs settled down after 15 days in NFs in pure water, methanol, ethanol, ethylene glycol, and 1-hexanol fluids at rates of 11, 25, 38, 18 and 47%. Kim *et al*.^17^ added three types of surfactants Sodium dodecyl sulfate (SDS), poly(propylene glycol)-block-poly(ethylene glycol) (P123) and triton X-100 to NFs containing 0.05 wt % RG (reduced G) in water and observed that, after a month, an NF containing triton X-100 was more unstable in comparison with the other ones. Shahrul *et al*.^18^ found that, after 90 min of ultrasonic stirring, water-magnetite NFs become unstable and deposited within a few min. Innovative designs of different types of stable ferrofluids (magnetic or ferroelectric NPs) dispersed in a suitable carrier liquid are well described with well-known chemical synthesis routes, and basic physics of functionalized medicals, biosensors, and other applications^19,20^. In order to investigate the surface modification of G on its stability, Uddin^20^ conducted a study in GO that was reduced using hydrazine monohydrate at 100 °C. That hardly disperses in a water-based NF and rather settles down rapidly. Askari *et al*.^21^ added cetyltrimethylammonium bromide (CTAB), triton X-100, and terpolymer surfactants to a water-nanoporous-G NF and observed that NPs had deposited after 3 days in the CTAB present. The NPs in the triton X-100 and terpolymer NFs deposited after two weeks and two months, respectively. Dehkordi *et al*.^22^ found that an increasing surfactant concentration in the base fluid alters its structure so as to result in foam and bubble formation, reducing the conductivity of the NFs. The additives finely modulate local structure and rheology in the NFs^23^.

Reviewing the literature on different kinds of NFs and studies of effects of an electric field on their thermophysical properties reveals that thermophysical properties of NFs could be improved through blending MNPs, and applying an electric field^24–28^. This can be described in Newton’s differential equations for such NPs under an electric field^29,30^

1 $${m}_{p}\frac{d{u}_{p}}{dt}={F}_{EHD}-{F}_{d}-{F}_{wp}-{F}_{ps}+{F}_{TS}$$

2 $${F}_{EHD}=qE-1/2{E}^{2}\overrightarrow{\nabla }\varepsilon +1/2\overrightarrow{\nabla }\left[{E}^{2}\rho {\left(\frac{\delta \varepsilon }{\delta \rho }\right)}_{T}\right]$$

3 $${F}_{d}=6\pi \mu R(\overrightarrow{{u}_{f}}-\overrightarrow{{u}_{p}})$$

4 $${F}_{wp}={m}_{p}g$$

where ${F}_{EHD}$ is the electrohydrodynamic force, ${F}_{d}$ is the drag force, ${F}_{wp}$ is the NP weight induced force, ${F}_{ps}$ is the force exerted on the NP by its upstream species, ${F}_{TS}$ is the force exerted by the underside fluid of the NP, ${m}_{p}$ is the NP mass, ${u}_{p}$ is the nanoparticle velocity, ${u}_{f}$ is the velocity of the fluid of density ρ, q is the electric charge, E is the electric field, ε is the dielectric permittivity, μ is viscosity and R is the radius of the NPs at temperature T.

This study uses the hydroelectrodynamic force (as Eqs. 1 and 2 show) as a controlling force to produce a G-based NF. For this purpose, we investigate the effects of various factors on deposition velocity and G-behavior in a water-ethylene glycol hybrid base fluid with a 60%/40% combination from a molecular perspective. Furthermore, the parameters of stabilizing such NFs by adding MNPs and applying an electric field are studied.

## Modeling

In this study, all simulations were conducted using the LAMMPS (large-scale atomic/molecular massively parallel) simulator, and the images were created utilizing an Ovito software^31^. The ReaxFF potential was used to simulate the intermolecular forces, angles, and charge equilibrium of the system^32^. The ReaxFF potential calculates the force exerted on an atom at each time step using the bond order or bond distance method introduced by Tersoff^33–36^,

5 $${E}_{system}={E}_{bond}+{E}_{over}+{E}_{under}+{E}_{lp}+{E}_{val}+{E}_{tor}+{E}_{vdWaals}+{E}_{Coulomb}$$

where the terms are the contributions of bond-energy, over coordination, undercoordination, lone pair, valence angle and torsion, Coulombic non-bonding and van der Waals energy, respectively. The potential of ReaxFF could be regarded as a bridge between molecular dynamics and quantum computation, which can be used to investigate complex systems.

**Figure 1:**
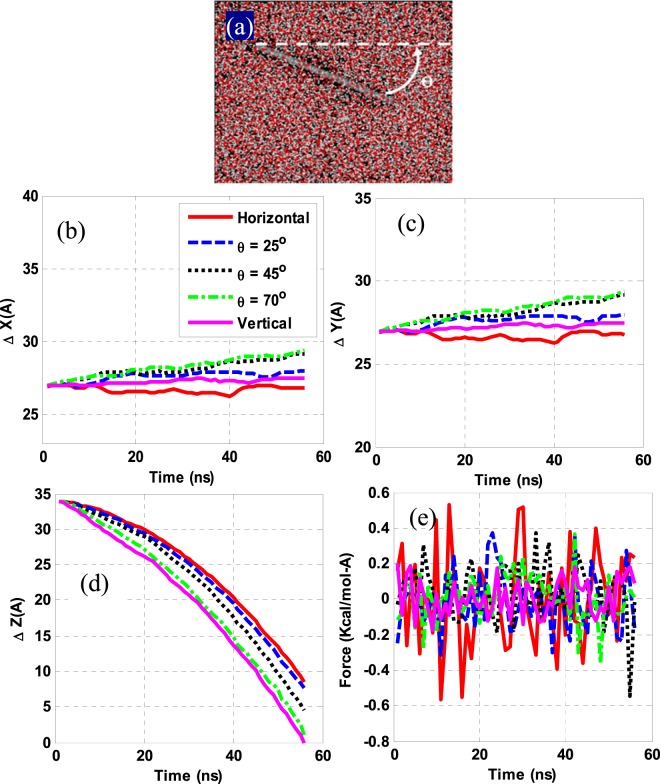
(**a**) Initial configuration of a G containing system, (**b–d**) average displacements (∆) of the G-sheet along different axes at different angles of inclination, and (**e**) average force applied to the G-sheet in the x-direction.

In this paper, the effects of angle of G-sheets in a dispersive phase in an NF, its temperature, the weight fraction of G by resizing, and the number of G-layers added to a base fluid are studied with an example of hybrid water (60%)-ethylene glycol (40%) heat transfer fluid. Besides, the feasibility of its stabilization was studied by adding MNPs and applying an electric field E. All systems were subject to constant pressure and volume to reach an equilibrium of 300 ps and then 700 ps, respectively. The simulation process was then pursued by applying a gravitational force of 2.2 Newton (N) with a time step of $1\times {10}^{-17}$ s under a fixed-energy ensemble. If the gravity acceleration applied to the system is assumed as $9\frac{{\rm{m}}}{{s}^{2}}$, due to using the exact ReaxFF potential utilization, which is the most accurate, yet the most time-consuming potential, the computational cost may considerably be increased and the simulation process may take months and even years to accomplish. On the other hand, because the purpose of this study is to juxtapose the effects of the above-mentioned factors, it is feasible to curtail simulation time via an optimal simulation time selection to achieve the objectives of this study. Numerous and pre-designed simulations reveal that owing to a great deal of gravitational force applied to the systems, the molecules do not have enough time to interact with one another and the G-moves down as a rigid object. Having a gradually decreased gravitational force, the optimal amount was found to be $5\times {10}^{17}\frac{m}{{s}^{2}}$, which could be attributed to a range of forces exerted on the NPs at the ReaxFF potential. Therefore, this value is considered as an optimal virtual acceleration for all simulations. Figure 1a illustrates a way G is placed in NFs at different angles to the horizon. The angles studied in this research were 0°, 25°, 45°, 75° and 90° respectively.

Additionally, to study the effects of G-fraction, a basic fluid structure with different modes of monolayer and multilayer G was studied. All primary structures include 4800 water molecules and 929 molecules of ethylene glycol as base fluid and G with a size of 3.5 nm × 3.5 nm at 30 °C. To study the effects of the G-content, first, the dimensions of the single-layer G-plates were increased and then the effects of fraction changes with an increasing number of layers were considered. To investigate temperature effects on G-deposition, the system was designed to contain a monolayer G with a size of 3.5 × 3.5 nm separately equilibrated at temperatures of 30, 45, 60 and 75 °C.

## Results and discussion

### The angle of G sheets

Figure 1(a) shows the G-sheet average displacement changes in the z-direction at different angles. A glance at Fig. 1(b) reveals that by increasing the angle of G from 0° to 90° in the fluid medium, its sitting rate increased so as it was deposited faster over a specified time interval, meaning that the NF produced becomes unstable. The observed delay in its deposition at zero degree could be attributed to a continuous shell creation by intermolecular forces of the underlying G- fluid, which promotes an interfacial tension therein.

The surface tension of the G is reduced by tilting its orientation so as it deposits faster. In other words, a zero-degree of its orientation creates a continuous shell of fluid molecules on the XY plane, which would be in contact with its largest possible area and thus creates the strongest resistance against its sedimentation. On tilting its orientation angle, the shell loses its contact area until its position to 90°, creating a surface tensile phenomenon on the YZ plane, as illustrated in Fig. 1c. This restricts the G-movement in the x-direction. As per the results in Fig. 1e, on a tailored surface tension in the YZ plane, the average force applied to the G- sheet in the x-direction at 90° changes gradually compared to other angles, which prevent the abrupt shift in the magnitude of the force in the x-direction. As illustrated in Fig. 1b, the G sitting at larger angles is parabolic; larger the angle smaller is its radius of curvature. The nonlinearity of this graph, in particular at zero degrees, indicates that the force is discontinuously applied to G-atoms. Examining G-behavior in the fluid suggests a wrinkle phenomenon as the main factor in this example. A mean G-displacement along the x and y axes {Fig. 1(c,d)} implies that the wrinkles have higher frequency and amplitude along with these directions.

The mean displacement is rather increasing in the x-direction for the G-sheet at a 70° angle on a decreasing surface tension over a reduced contact area. This makes the atoms at the G’s most rear row readily break down the interfacial forces and penetrate the base fluid more easily, leading to be depositing faster compared to that at larger angles. As mentioned earlier, in the 90° case, the surface tension on the YZ plane impeded the free G-movement in this direction. The curvature radius, as on diminished in the said angle is enhanced, reflects in a drop in the wrinkle effect through rising this angle. As this angle is raised up, a decreased surface tension eases the G settle down more rapidly rather than forming a wrinkle.

### Effects of G-contents

#### Effects of G-contents by resizing single-layer G-sheets

**Figure 2:**
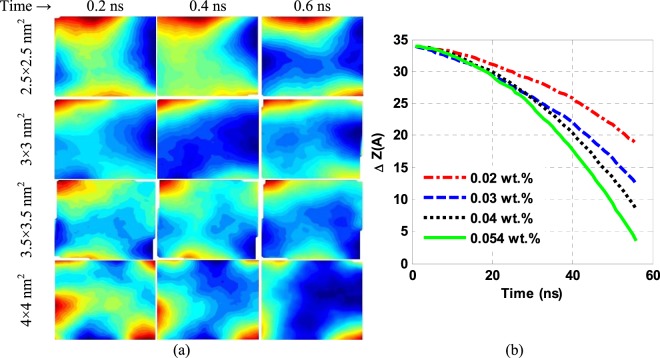
(**a**) Position of C-atoms in the G-sheets for the considered sizes at different times and (**b**) mean displacements for various contents of the G-sheets of different sizes.

To investigate the effects of G-fraction by resizing the sheets, four samples containing G of 2.5×2.5 nm^2^, 3×3 nm^2^, 3.5×3.5 nm^2^, and 4×4 nm^2^ sizes were prepared and studied. According to Fig. 2a,b, by increasing the G-fraction and its size in the sheets, the G-sitting rate increased in a given time interval in the NF becomes more unstable. Looking further at the G-sheet average displacement plots in Fig. 2b, it is observed that these are nearly linear at lower sizes and small contents of the G-sheets in the NFs in parts of a parabolic trajectory. The curvature is grown in these parabolic paths in the later samples. In G-content increased from 0.02 to 0.03, 0.04, or 0.054 wt%, its saturation rate is enhanced by 43, 82, or 109%, respectively. Evidently, the G-sitting rate is not linearly dependent on its content in the NFs.

In principle, a corrugation of a G-surface can be divided into three categories: wrinkle, ripple, and crumple, where the aspect ratio of the wrinkle is higher than that of ripple and its height is less than 15 nm^37^. Fig. 2a illustrates the position of C-atoms in G-sheets in four NFs prepared with various G-sizes at different times. The visible phenomenon on the G-surface in the first NF so obtained is a ripple, in which the G-atoms shift downward with the least off-plane deformation. In a larger G-size, it converts into a wrinkle and amplitude of this deformation grows, leading to a rise in the penetration of the G-atoms into the sub-G fluid, which, in turn, disrupts an interconnected network of the fluid.

As noted above, a decrease in the surface tension promotes the G-sitting rate in a non-linear relationship of it over its effective contents. Here, as the G-content rises-up on increasing the G-size and as the prepared NFs contain the same base-fluid in a fixed number of molecules, more base-fluid moieties would trap in G-sheets of larger sizes. In other words, in this case, G-could is considered as a barrier to the movement of the base-fluid entities on reduced freedom of local motion, resulting in an intermolecular force growth. As a result, the surface tension under G rises-up and leads to a nonlinear relationship between its content and sitting rate. However, a growing wrinkling overcomes the increasing surface tension and, ultimately, the G settles down faster in the NFs over its increasing sizes.

### Effects of G-contents by varying the number of G-layers

**Figure 3:**
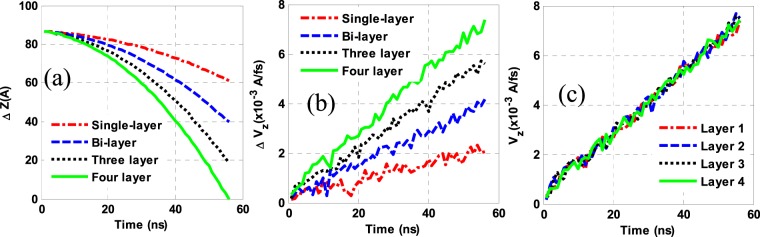
(**a,b**) The average displacement and velocity changes (∆) of the first layer of G-sheets and (**c**) the velocity of different layers of G in a system with four-layer G-sheets.

To investigate the effects of G-contents by increasing the number of G-layers, the samples containing one, two, three, and four G-layers were considered. Figure 3(a,b) illustrates the mean displacement and velocity of the first G-layer for the various states studied. An overview of these plots indicates that as long as the added size and mass of the G-layers to the base fluid are fixed, the G- sitting rate rises as the number of G-layers increases. Further, adding one, two and three G-layers to a monolayer G, the G- sitting rate grows by 77, 153, and 263%, respectively. These values indicate the importance of a monolayer-G in this example. However, many monolayer G-sheets in the market have a significant fraction of a multilayer-G, and therefore, in some cases, the addition of G to a base fluid deteriorates its thermophysical properties, which could be attributed to its settlement in a modified NF.

**Figure 4:**
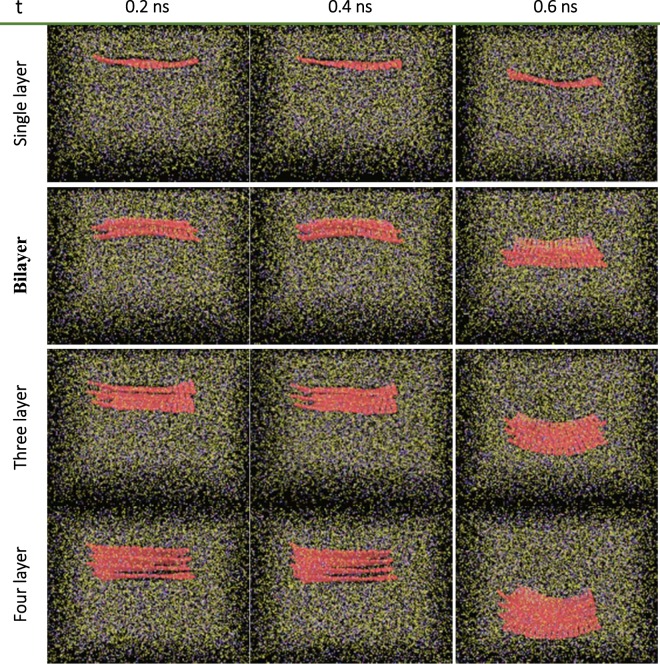
G-behaviors in NFs containing monolayer, bilayer, three-layer and four-layer G at t = 0.2, 0.4 and 0.6.

As portrayed in Fig. 4, examining the G-behavior in the various NFs reveals that an increasing number of G-layers reduces the transition time from wrinkle to bulk while the depth of deflection in the last G-layer has grown-up. Since the number of fluid molecules and size/weight of the G-layers used here are fixed, an increased sitting rate and the amplitude of deflection created in the G-sheets are their inherent properties.

As mentioned earlier, G has unique properties of its hybrid sp^2^ orbitals in which the bonds in the orbitals overlap head-to-head are rich in the electron density along the bond axis, so-called σ-bonds. Electrons in unhybridized C-orbitals in the other bonds form π-bonds in the side-by-side (edgewise) overlap of the parallel p orbitals. As less overlapping than σ-bonds, the π-bonds are rather weaker^38^. Zhang *et al*.^39^ showed that the binding-energy, for a specific radius of curvature, arises-up as the number of G-layers growing and it promotes their bending strain. A monolayer can easily curve only by introducing a π-orbital misalignment between adjacent pairs of C-atoms. However, in the case of a multilayer, the van der Waals forces mediate the load transfer between layers in its bending involves extension or compression of a σ-bond. Thus, as the number of G-layers increases, the effects of σ-bonding are strengthened. This, in turn, enhances the amplitude of the valley created in the G-surface, which leads to more G-layers entering the fluid under G. Such growth reduced the surface tension and fluid resistance to the G-sitting, resulting in its faster deposition in this example.

Figure 3c illustrates the velocities of different G-layers in a sample with four-layer G-sheets. Since the G-sitting speeds of the different layers are practically equal, it could be concluded that the σ-bonds are strong enough to evenly interbond the G-layers one over others. It seems that the different G-layers are completely interconnected, very much similar to a G-sheet of 4 times the weight of a single-G-layer with the same size and equal gravitational force. This will promote the net weight force and further instability in an NF. However, as the surface tension depends on the size of the G-sheet and as long as the NFs studied here contain G-sheets of the same size, the contact area retains the same value, and thus a resistive force applied to the G-fluid surface would not vary as a result of an increasing G-fraction due to a rise in the number of its layers. As a result, with the resistive force remaining constant in this case, a nearly linear relationship extends between the weight fraction of G and its sitting rate.

### The effect of temperature change

**Figure 5:**
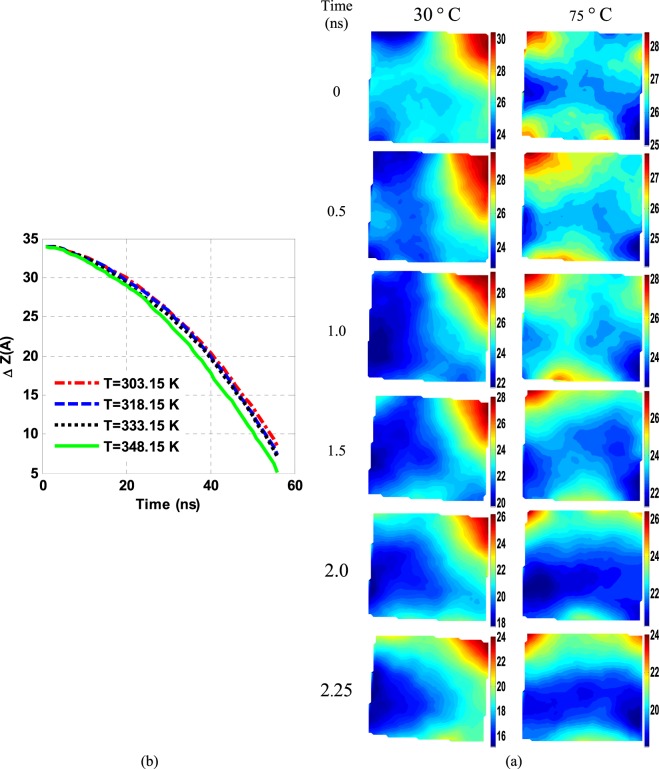
(**a**) Average displacements (in Å) of the G-sheets, and (**b**) the position contours of C-atoms at different time intervals and temperatures.

Figure 5a illustrates the average displacement of the G-sheet in the z-direction at different temperatures over a time scale. As the temperature rises over a certain period, more and more G settles down. A G-sitting rate has increased by 7.5% by raising a temperature of 45 °C from 30 °C to 75 °C. The due position changes in the G-sheet are described in Fig. 5b at the two temperatures. Taking these results into account, it could be observed that as the temperature rises, the wrinkle wave propagation velocity grows on the G-surfaces. Such behavior could be due to increased mobility of the G-atoms and thus a duly decreased surface tension. The wave propagation velocity rises-up on the G-surface leaves insufficient time for the base fluid molecules to rearrange themselves. On the other hand, as the NF warming up, the local mobility of the base fluid molecules rises up accordingly, resulting in a due resistive force reduction against the G-sheet and hence a faster G-deposition.

### Effects of MNPs and applied electric fields

**Figure 6:**
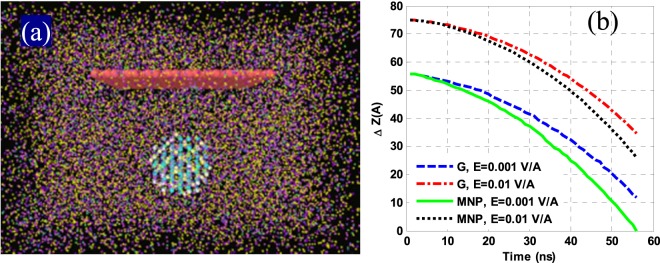
(**a**) Primary structure of a water/ethylene glycol-G NF containing MNPs and (**b**) average displacements of G/magnetite (M) atoms under the electric field along the z-axis.

To study the hypothesis of stabilizing NFs by adding MNPs (magnetite) in an applied electric field, MNPs (of a diameter 16 Å) were added to an NF of an ethylene glycol/G in water, where the G-sheet was placed horizontally (Ө = 0), at an initial 25 °C temperature. The basic structure of the NF is shown in Fig. 6a. As described in Fig. 6b, it is observed that a stable NF could not be created in an applied electric field as large as 0.001 to 0.01 V/Å. Here, the result of the unsteady forces of Eq. (1) is greater than the electrohydrodynamic force. Thus, the applied field merely reduces the rate of G-sitting in the G could not suspend in the dispersing phase.

**Figure 7:**
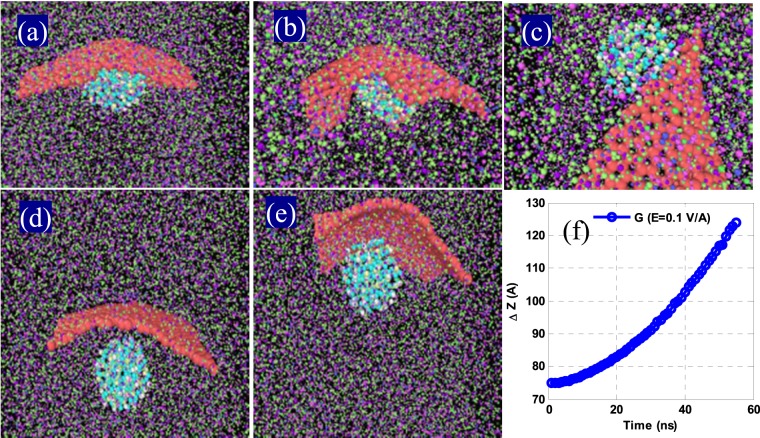
(**a**) The moments of nanoparticle collisions with G-sheets, (**b**) the motion of G as a shell on a magnetite core, (**c**) the MNP separated from the G-shell, (**d,e**) behaviors of magnetite and G-NPs under a 0.1 V/Å electric field at two different time intervals, and (**f**) average displacement of the G-sheet in the electric field.

Applying an electric field as large as 0.5 V/Å, MNPs overcome the intermolecular forces of the base fluid, rapidly approaching and colliding with the G, creating a G-shell on the surface of the MNPs. Considering the Columbus forces are larger over all the resistive forces, the G-binds over the MNPs in a core-shell. However, as the number of G-atoms is not same on either side of the magnetic core, it exerts a net upward force on the G-side of the NP and, on the opposite side, the downward weight forces of G-atoms create a torque on the G and let G-shell detach from the surface of the MNPs over a due time interval, causing them to coagulate in the upper part of the NF. This eventuates in a dramatic instability of the NF as described in Fig. 7.

It is observed that in an electric field of 0.1 V/Å, the NPs no longer overcome completely the intermolecular forces of the base fluid and rapidly adsorb over a nearby G-sheet. As a result, a G-shell is not seen on the surface of MNPs. However, as soon as the Columbus forces exceed the resistive forces, upward pressure is built up at the contact surface of the fluid, leading to coagulation of the NPs in the upper part of the sample of an unstable NF.

**Figure 8:**
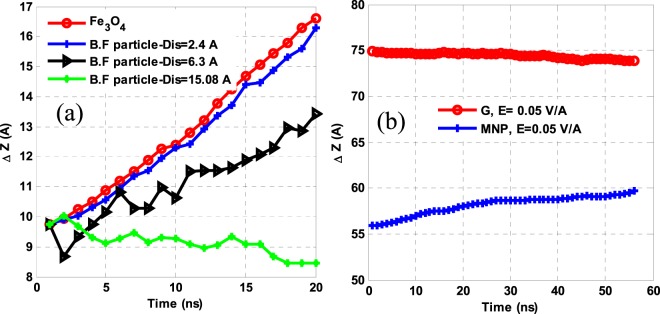
(**a**) A trajectory in the NPs and base fluid move at different positions around the NPs over a time scale and (**b**) the mean displacements of G and MNPs along the z-axis.

To investigate the interaction of base fluid molecules with MNPs, an atom at the NP surface, as a reference point, and molecules inside the base fluid at δ = 2.46, 6.30 and 15.08 Å were randomly selected. Figure 8a displays the position of the selected atoms over a time scale in the z-axis. The positions of the base fluid molecules located close to the MNPs are strongly dependent on the positions of the NPs and such dependency breaks down sharply as the separation increases. At δ = 15.08 Å, the behavior of the underlying fluid molecules is independent of the NPs behavior. A tailored fluid dynamics in the NPs present could be attributed to an electrostatic interaction between them on induced electrostatic charges on the NPs surfaces and the fluid moieties - in a manner that as the separation increases the electrostatic force decreases and ultimately vanishes. Given that the interaction between the NPs and the base fluid is via electrostatic forces, a closer examination of the NPs behavior under the electric fields of 0.5 and 0.1 V/Å reveals that the MNPs serve as a bridge between the fluid molecules. In other words, as the applied electric field rises, the force applied to the NPs rises-up and the NPs overcome the intermolecular forces of the base fluid and travel upwards. Because of the electrostatic force between the NPs and the dispersing phase molecules, the base fluid molecules collide around the MNPs and since the NPs displace quickly when the electric field is intensified, the remaining molecules do not get any chance to rebond so quickly.

**Figure 9:**
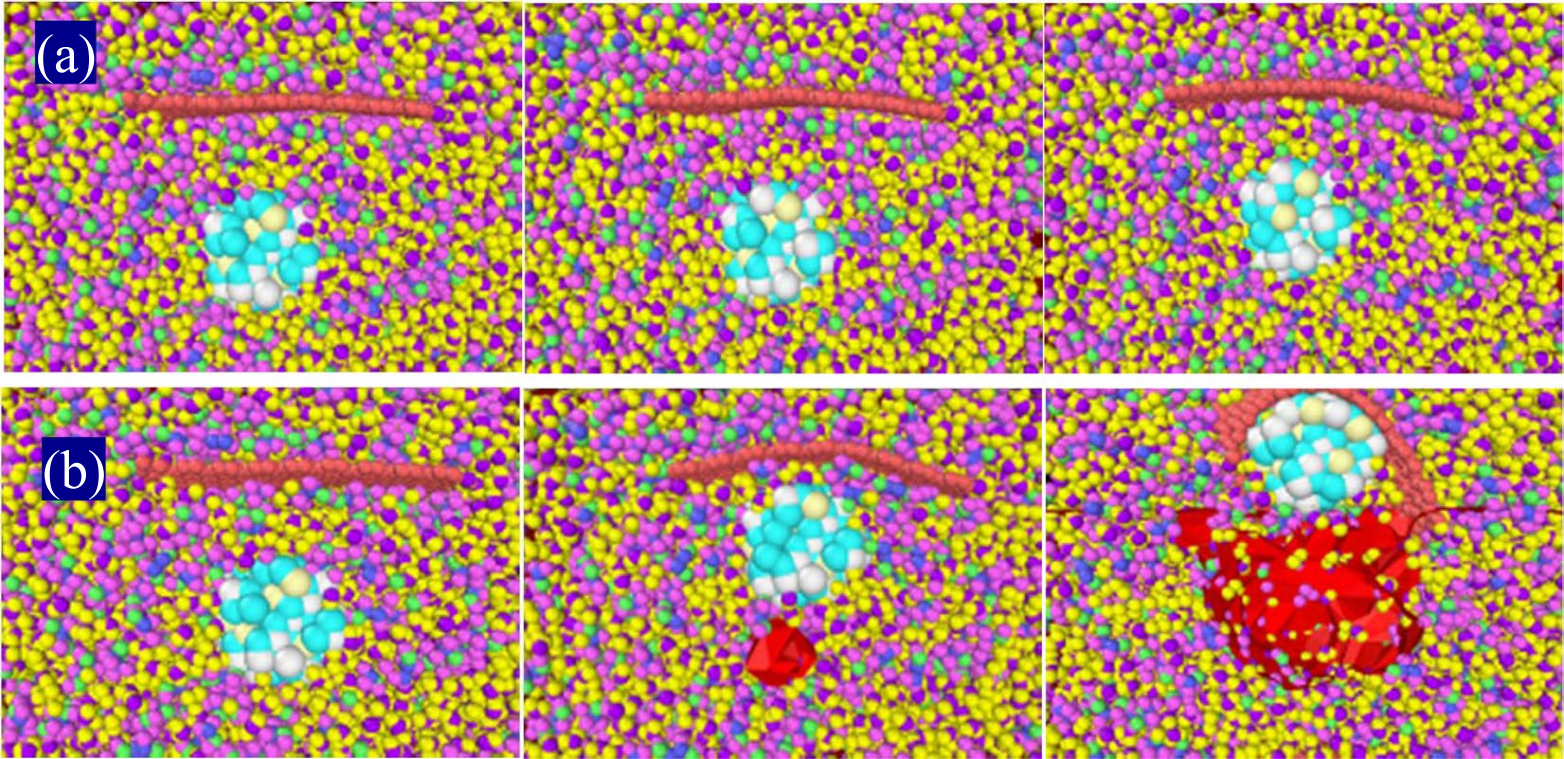
The Breakout view of the NF container center in an applied electric field (**a**) 0.1 V/Å and (**b**) 0.5 V/Å at t = 0, 0.2, and 0.52 ns. An empty cavity created in the NF (marked in red color) accumulates around the NP surface by electrostatic force.

As a result, a local displacement of the NPs creates an empty cavity in a dispersed phase, while the application of a weaker electric field causes the fluid around the NPs to gently rebond one another in a network, displacing the NPs upward and reducing the average electrostatic force. Therefore, a vacuum cavity no longer forms within a dispersing phase in a stable NF, as described with the models in Fig. 9. As shown in Fig. 8b, an applied electric field 0.05 V/Å balances a sum of the unsteady and resistive forces in Eq. (1), and thus both G and MNPs retain to be suspended in a stable NF. This critical limit of the electric field equilibrated here is calculated separately for each problem in this investigation.

## Conclusions

In this paper, the factors affecting the stability of a water/ethylene glycol NF containing G-sheets are studied by reactive molecular dynamics, wherein as the G-angle on the base fluid grows, the effects of G-surface tension decreasing until the G is oriented up to 90° in the base fluid. The studies of G-behavior at different angles suggest that a wrinkling phenomenon creates at a zero-degree angle of orientation toward the z-axis in a resistant factor against G-settling. As the size of the G-sheet increases, a corrugation created on the G-surface changes from a ripple to the wrinkle. As a result, the G settles faster and causes NF instability. On increasing the number of G-layers, the resistance force against the G-settling eventually shrinks and results in a more unstable NF. A nonlinear relationship is observed between the G-sitting rate and G-content owing to an increase of its effective sizes. NFs produced by higher G-contents through an increasing G-size appears to be more stable than those created by an increasing number of G-layers. As warming the sample, the velocity of wrinkle movement arises on the G-surface as on the fluidity increases in the fluid, resulting in a decrease in the intermolecular forces on the bottom of the G-layers, which ultimately eventuates in a poor NF stability. Finally, an electrohydrodynamic force can be employed as a controlling force to stabilize NFs and control NPs dynamics in a manner that through adding MNPs and applying an electric field, such as 0.05 V/Å, a water/ethylene glycol/G NF could be completely stable.

## Supplementary information

Supplementary Video.
